# Optimization of a Low Surface Energy Coating for Enhanced Water Resistance and Condensation Suppression

**DOI:** 10.3390/ma17215238

**Published:** 2024-10-28

**Authors:** Siwei Pan, Yanwen Ouyang, Yaohong Zhao, Qing Wang, Yihua Qian, Chunqing He

**Affiliations:** 1 Electric Power Research Institute of Guangdong Power Grid Co., Ltd., Guangzhou 510080, China; siweipan@163.com (S.P.); kennyaoo@126.com (Y.Z.); sdytwangqing@163.com (Q.W.); 13926035192@163.com (Y.Q.); 2 School of Physics and Technology, Wuhan University, Wuhan 430072, China; 2019102020078@whu.edu.cn

**Keywords:** low surface energy, water resistance, anti-condensation, orthogonal experimental design

## Abstract

This study focuses on formulating a low-surface-energy, water-resistant, and anti-condensation coating utilizing a fluorocarbon and acrylic resins composite (FAC), enhanced by six functional additives: antistatic agents, water-repellent agents, nanofillers, anti-mold and anti-algae agent, leveling agents, and wetting and dispersing agents. An orthogonal experimental design was implemented to systematically investigate the effects of varying concentrations of these additives on the surface tension of the coating. The results show that the optimized combination of fluorocarbon and acrylic resins composite (OFAC)with functional additives significantly reduces the surface tension, thereby improving both water resistance and anti-condensation properties. This research advances the development of more efficient surface treatment technologies, particularly for applications requiring enhanced water resistance and anti-condensation performance.

## 1. Introduction

In recent years, multifunctional coatings with both water resistance and anti-condensation properties have become increasingly important across various industries, such as construction, electronics, and automotive sectors [1,2,3]. These coatings primarily serve to protect surfaces from moisture infiltration and condensation, preventing corrosion, material degradation, and potential malfunctions in sensitive equipment [4,5,6,7]. Therefore, developing coatings that can effectively repel water and reduce surface condensation is crucial for enhancing material durability, extending service life, and ensuring stable performance in harsh environments.

The key to achieving these functionalities lies in reducing the surface energy of the coating [8,9,10]. Low-surface-energy coatings work by preventing water droplets from spreading across the surface, instead causing them to form spherical beads that easily roll off, a phenomenon known as the “lotus effect” [11]. This significantly enhances water resistance by minimizing the contact area between the water and the surface, reducing the likelihood of water penetration [12]. Moreover, lowering the surface energy also inhibits the condensation of water vapor into droplets, which is particularly important in environments susceptible to temperature fluctuations or high humidity [13]. By selecting materials with inherently low surface energy, it is possible to create coatings that not only repel water but also prevent the formation of condensation. This dual functionality is especially valuable in climates where large temperature variations often result in condensation buildup.

Fluorocarbon resins are renowned for their low surface tension, making them ideal for superhydrophobic coatings [14,15,16]. The small size and high electronegativity of fluorine atoms form strong carbon–fluorine bonds, effectively reducing surface tension and enabling these resins to repel water and oil while providing resistance to chemicals, weathering, and thermal degradation [17]. Liu et al. successfully constructed a waterproof coating using fluorosilicone resin as a binder and modified hydrophobic carbon nanotubes to build a micro-nanostructure [18]. Wang et al. inhibited the surface charge accumulation of the resin by covering 1,1,2,2-tetrahydroperfluorodecyltrimethoxysilane on the surface of alumina and using it as a functional additive [19]. The coatings exhibited enhanced water-repellency properties due to the special properties of perfluorooctyl chains on the microscopic scale and the intricate morphological structure on the mesoscale. Yang et al. synthesized a series of anhydride-based polypropylene (MPA) resins with different substituents (R) in their side chains by free radical polymerization of (meth)acrylate, styrene, and maleic anhydride [20]. A low surface energy MPA coating was designed by combining long-chain alkane groups with fluorocarbon groups. The optimized coating with a molecular ratio of 0.8:0.4 of stearyl methacrylate to 1H,1H,2H, and 2H-heptadecafluorodecyl acrylate exhibited water resistance. However, despite their properties, pure fluorocarbon resins may exhibit limitations in terms of adhesion, which can reduce their effectiveness in certain applications. To address this, modifying fluorocarbon resins with acrylics [20,21,22] enhances adhesion, water resistance, and durability, creating composites that perform well in both water-resistant and anti-condensation applications. This modification ensures that coatings can bond effectively to various surfaces while maintaining their protective properties over time. In addition to the base resins, functional additives play a crucial role in enhancing the performance of coatings. Commonly used additives include water-repelling reagents, nanofillers, antistatic reagents, anti-mold and anti-algae reagents, leveling reagents, and wetting and dispersing reagents [23,24,25]. These additives enhance the coating’s water resistance, antistatic properties, mechanical strength, durability, and overall uniformity and surface quality.

This study focuses on preparing a low surface energy, water resistant, and anti-condensation coating using a composite matrix of fluorocarbon and acrylic resins (FAC) optimized with the addition of six functional additives. A systematic investigation employing an orthogonal experimental design was carried out to assess the effects of varying additive concentrations on the surface energy of the coating. The objective was to optimize the additive formulation to reduce surface energy and enhance the water resistance and anti-condensation properties of the coating. The results from this study offer significant knowledge for the development of more efficient coatings for applications requiring improved surface protection.

## 2. Materials and Methods

### 2.1. Experimental Materials

The experimental materials used in this study consist of two types of resins: fluorocarbon resin (ETERFLON 41029) from Eternal Materials Co., Ltd. (Taiwan, China) and acrylic resin (FS-4470) from Elementis Dekian Chemical Co., Ltd. (Shanghai, China). The curing agent employed was BAYER N3390 from Bayer AG (Leverkusen, Germany). The solvents, including butyl acetate and propylene glycol methyl ether acetate (PMA), were supplied by Tianjin Bodi Chemical Co., Ltd. (Tianjin, China). Additionally, six functional additives were utilized: a water-repelling agent (4301) from Wuhan Changpu Technology Co., Ltd. (Wuhan, China), an anti-mold and anti-algae agent (copper pyrithione) and antistatic agent (QCS) from Shanghai Yuanye Biotechnology Co., Ltd. (Shanghai, China), as well as nanofiller (titanium dioxide), leveling agent (TEGO-300), and wetting and dispersing agent (TEGO-871) from Evonik Industries AG (Essen, Germany).

### 2.2. Preparation of Coating

Firstly, the clearcoat was prepared by mixing fluorocarbon and acrylic resins; specifically, 40 g fluorocarbon resin and 4.5 g acrylic resin were added to a mixture of 30 g PMA and 20 g butyl acetate and stirred for 30 min. The additives were added according to an orthogonal design, and a six-factor, three-level orthogonal design was used in this experiment, as shown in Table 1. The six factors refer to six functional additives: water-repelling reagent, nanofiller, antistatic reagent, anti-mold and anti-algae reagent, leveling reagent, and wetting and dispersing reagent. Three different concentration levels were set for each additive, i.e., low (level 1), medium (level 2), and high (level 3). The concentration combinations of the additives were arranged according to the orthogonal experimental scheme, as shown in Table 2, which ensured a uniform distribution of the levels of the factors in the whole experiment, thus reducing the number of tests and optimizing the coating formulations.

After the functional additives were thoroughly mixed with the resin solution, a 4.45 g curing agent was added and stirred for 30 min to obtain the coating. The prepared coating was then uniformly applied to the substrate using a spraying technique with a spray pressure of 0.3 MPa and a distance of 15 cm between the nozzle and the substrate. The coated samples were allowed to dry at room temperature for 10 min and then dry in an oven at 80 °C for 30 min.

### 2.3. Testing and Characterization

#### 2.3.1. Surface Characterization

The surface morphology of the coating was analyzed using scanning electron microscopy (SEM, S-4800, Hitachi, Tokyo, Japan). The SEM was operated at 10 kV accelerating voltage, 20 μA beam current, and 9 mm working distance.

The surface energy of the coating was determined using the contact angle method, which is derived from the principles of Young’s equation. This method is one of the most widely adopted techniques for surface energy measurement. The surface energy of the solid ($\gamma_{s}$) is related to the surface energy of the liquid ($\gamma_{1}$) and the contact angle (θ) through the equation:

(1) $$\gamma_{s}=\gamma_{s1}+\gamma_{1}\cos\theta$$

As described by Van Oss [26,27], the surface energy can be divided into two components: the Lifshitz–van der Waals component ($\gamma^{LW}$) and the Lewis acid–base component ($\gamma^{AB}$):

(2) $$\gamma =\gamma^{LW}+\gamma^{AB}$$

The Lewis acid–base component ($\gamma^{AB}$) can be further split into acid ($\gamma^{+}$) and base ($\gamma^{-}$) parameters, which are expressed as:

(3) $$\gamma^{AB}=2{\left(\gamma^{+}\gamma^{-}\right)}^{1/2}$$

To perform the measurements, at least one nonpolar liquid and two polar liquids were used, with known values for their surface energy components ($\gamma^{LW}$, $\gamma^{+}$, and $\gamma^{-}$), as provided in Table 3.

(4) $$\gamma_{1}(1+cos\theta )-2{\left(\gamma_{S}^{LW}\gamma_{1}^{LW}\right)}^{1/2}=2{\left(\gamma_{S}^{+}\gamma_{1}^{-}\right)}^{1/2}+2{\left(\gamma_{S}^{-}\gamma_{1}^{+}\right)}^{1/2}$$

A contact angle meter (SL200B, Kono, MA, USA) was used to measure the contact angle for evaluating the surface energy of the coating. The test involved using distilled water, formamide, and diiodomethane as probe liquids, with each liquid representing different polarities. For each sample, 5 μL of each liquid was dropped onto the surface, and measurements were taken at three distinct points. The average contact angle from these three points was recorded as the final result for each liquid. The contact angles from the liquid pairs were used to calculate the surface energy of the coating based on the known surface energy components.

#### 2.3.2. Water Resistance Performance

**Figure 1:**
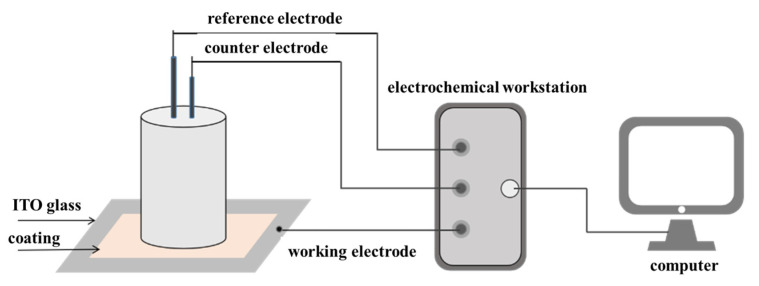
Schematic diagram of the three-electrode system for electrochemical impedance spectroscopy (EIS) testing setup.

To assess the water resistance of the coating, electrochemical impedance spectroscopy (EIS) tests were performed using an electrochemical workstation (CS310, Corrtest, Wuhan, China). The experimental setup, as shown in Figure 1, employed a traditional three-electrode system consisting of a working electrode, a reference electrode, and a counter electrode. The sample was prepared by spraying the coating onto a 3 cm × 2.5 cm conductive glass substrate. Once coated, the sample was positioned between the working and counter electrodes. A 3.5 wt.% NaCl solution was placed in a cylindrical container to serve as the electrolyte. The working area of the electrode was approximately 1 cm^2^. EIS measurements were carried out across a frequency range of 0.1 Hz to 105 Hz, with a perturbation voltage of 20 mV.

#### 2.3.3. Anti-Condensation Performance

The anti-condensation performance of the coating was tested in a climate chamber. A Peltier cooling plate was placed vertically, with the coated sample fixed onto it. The sample surface temperature was set to 8 ± 1 °C, and the chamber temperature and humidity were maintained at 20 °C and 85%, respectively. Sample temperatures below the surrounding dew point temperature caused condensation on the sample surface. This temperature difference induced condensation on the sample surface. Notably, based on the results of our field tests combined with data from the website of the Guangzhou Meteorological Service of China, this temperature effectively simulates a moderate cooling scenario during the fall and winter, allowing us to evaluate the performance of the coating in environments where condensation control is critical, such as in cooling systems or humid climates.

The condensation process was recorded using a high-speed camera (FASTCAM Mini Ux50 Type 160 k-M-8 GB, Leica, Wiesler, Germany) and a metallurgical microscope (DM2700MH, Leica, Wiesler, Germany). The camera’s frame rate of 60 fps and the microscope’s magnification of up to 200× capture the nucleation, growth, and development of condensate on coated surfaces. These high-resolution images allow detailed observation of the dynamic behavior of condensates on coatings.

## 3. Results

### 3.1. Wettability and Surface Morphology

As outlined in Section 2.2, an orthogonal design was employed, using the surface energy of the coating as the key evaluation metric. Table 4 presents the surface energy test results for 18 experimental samples, each prepared with varying concentrations of additives. It was observed that changes in additive concentration directly affect the surface energy of the coatings, highlighting the importance of optimizing the formulation to achieve the desired properties. To facilitate further analysis, Table 5 provides the mean values and ranges for each factor level, calculated based on the results from Table 4. The range is defined as the difference between the maximum and minimum mean values for each factor. From this data, it becomes clear that the water-repelling reagent has the most significant influence on the final surface energy, followed by the nano-filler. Other factors, while important, have a comparatively smaller effect. The factors influencing the coating’s surface energy are ranked in the order of A > B > C > F > E > D.

Based on the experimental results, the optimal formulation for the low surface energy coating is identified as A3B3C3D3E2F3, which corresponds to the following composition: 0.9 g of water-repellent agents, 6 g of nanofillers, 3 g of antistatic agents, 0.9 g of anti-mold and anti-algae agents, 0.6 g of leveling agents, and 0.9 g of wetting and dispersing agents. The coating prepared using this optimized formula of fluorocarbon and acrylic resins composite with six additives is referred to as OFAC.

**Figure 2:**
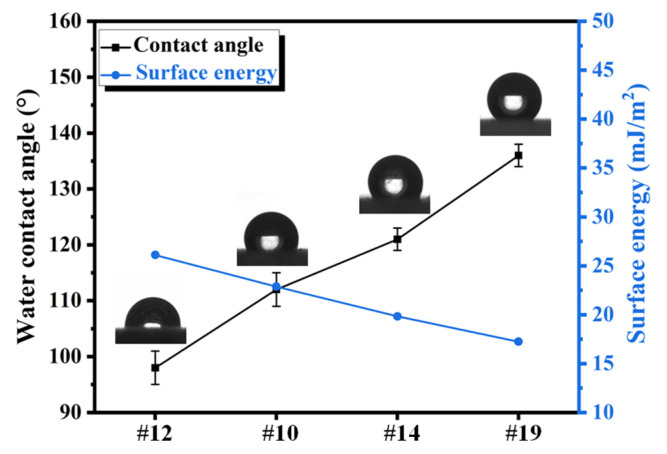
Water contact angles and apparent surface energies of coatings of Sample 12, Sample 10, Sample 14, and OFAC coatings, the insets are profiles of water droplets on the coating surfaces.

As shown in Figure 2, increasing the concentration of the water-repelling reagent and nano-filler TiO_2_ led to a significant improvement in hydrophobicity, with the water contact angle increasing from 96 ± 3° to 135 ± 3°. Correspondingly, the apparent surface energies of coatings No. 12, 10, 14, and OFAC were measured at 26.01, 22.87, and 15.86 mJ/m^2^, respectively, as is shown in Table 6.

**Figure 3:**
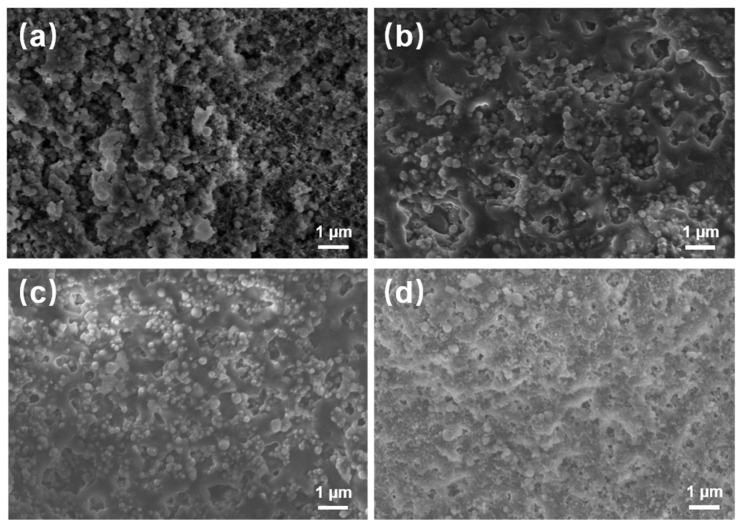
SEM images of as-prepared coatings with different additives of Sample 12 (**a**), Sample 10 (**b**), Sample 14 (**c**), and OFAC (**d**).

The superior liquid repellency observed on the surface of OFAC can be attributed primarily to its low surface energy and the presence of a micro-nano structure [28]. The surface morphologies of Samples 12, 10, 14, and OFAC are illustrated in Figure 3. When a water-repelling reagent (fluorosilane) and TiO_2_ nanoparticles are added to the original acrylic coating, the nanoparticles tend to aggregate, forming bumps, as depicted in Figure 3a. These protrusions increase surface roughness, resulting in a hybrid Wenzel-Cassie wetting state, which is consistent with previous findings [29,30]. As the weight percentage of water-repelling reagent (fluorosilane) and TiO_2_ nanoparticles increases, the coating becomes denser, with uniformly dispersed microcavities and nanopores (Figure 3d). These features serve as air-trapping sites, which enhance the coating’s hydrophobic properties.

### 3.2. Water Repellent Performance

**Figure 4:**
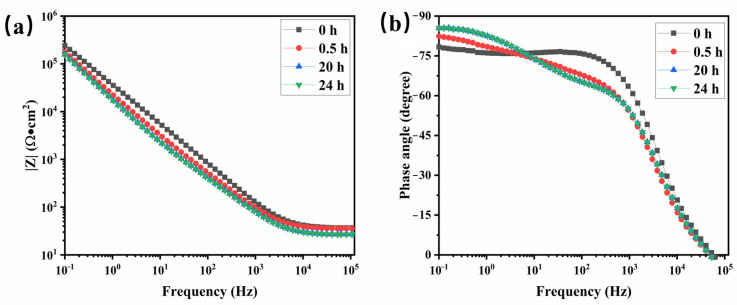
Electrochemical impedance spectra of clearcoat immersed in a 3.5 wt.% NaCl solution for various times. (**a**) the impedance magnitude curves, (**b**) the phase angle curves.

Electrochemical impedance spectroscopy (EIS) is employed to assess the water-repellent properties of organic coatings. The variation in impedance modulus over-soaking time, along with changes in the shape of the Nyquist and Bode plots, provides insight into the system’s performance and the kinetics of water diffusion within the coating. Figure 4 presents the electrochemical impedance spectra of the clearcoat at different immersion times. Throughout the soaking period, the impedance magnitude remains relatively stable, consistently maintaining a value of 10^5^ Ω·cm^2^ at 0.1 Hz. This stability indicates minimal diffusion of water molecules into the sample, suggesting that the resin forms a dense, cross-linked structure during curing, effectively preventing the penetration of water. As a result, the composite resin system demonstrates notable water resistance.

**Figure 5:**
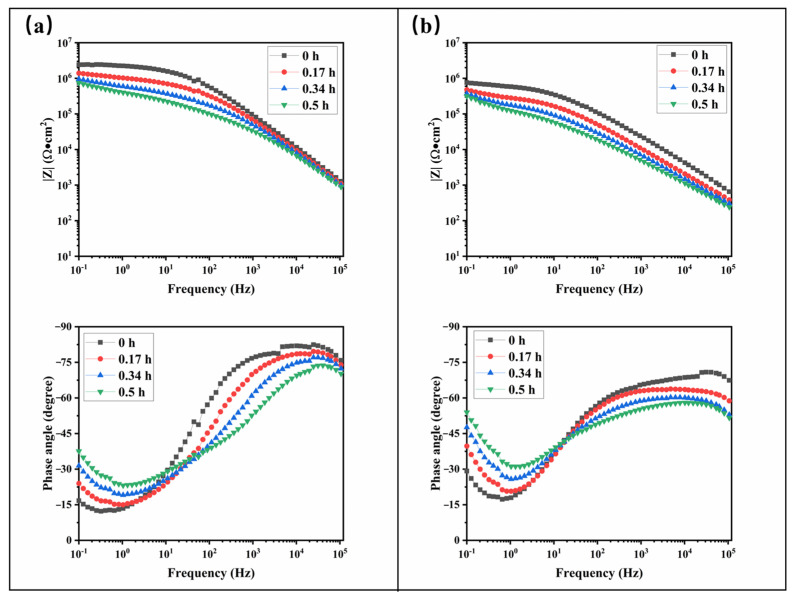
Electrochemical impedance spectra of samples 12 (**a**) and 10 (**b**) immersed in a 3.5 wt.% NaCl solution for various times.

Figure 5 presents the electrochemical impedance spectra of samples 12 and 10 immersed in a 3.5 wt.% NaCl solution over varying periods. Initially, both samples exhibit an impedance magnitude of approximately 10^6^ Ω·cm^2^ at 0.1 Hz. As immersion time increases, the impedance magnitude (|Z|) decreases, and the phase angle curves shift downward, indicating gradual solution infiltration and a corresponding reduction in the coating’s impedance. In the phase angle–frequency curves, within the first 0.5 h of testing, the phase angle approaches −45°, suggesting that the diffusion process follows Fick behavior, indicating a loss of water resistance. Furthermore, the frequency corresponding to the minimum phase angle increases progressively with immersion time, highlighting noticeable delamination of the coating after just 0.5 h of exposure to the solution.

**Figure 6:**
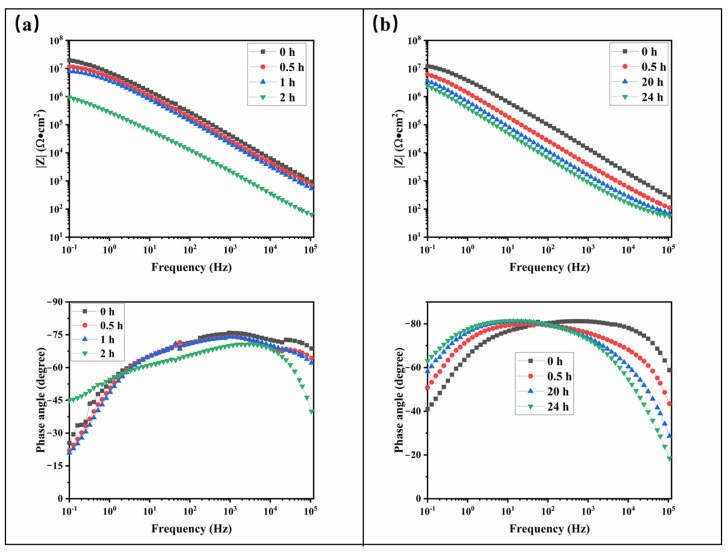
Electrochemical impedance spectra of samples 14 (**a**) and OFAC (**b**) immersed in a 3.5 wt.% NaCl solution for various times.

Figure 6 presents the electrochemical impedance spectra of samples 14 and OFAC as a function of immersion time in a 3.5 wt.% NaCl solution. Initially, as shown in Figure 6a, sample 14 exhibits an impedance magnitude of approximately 10^7^ Ω·cm^2^, indicating excellent water resistance. However, after 2 h of immersion, the impedance magnitude decreases by an order of magnitude to 10^6^ Ω·cm^2^, suggesting that the electrolyte solution has begun to permeate the coating through its pores.

Figure 6b demonstrates that during the early stages of immersion, the |Z| curve of the OFAC gradually shifts toward the low-frequency region. After 20 h of immersion, the impedance magnitude |Z| stabilizes, and even after 24 h, no second-time constant is observed, indicating that the coating maintains good water resistance despite extended exposure.

**Figure 7:**
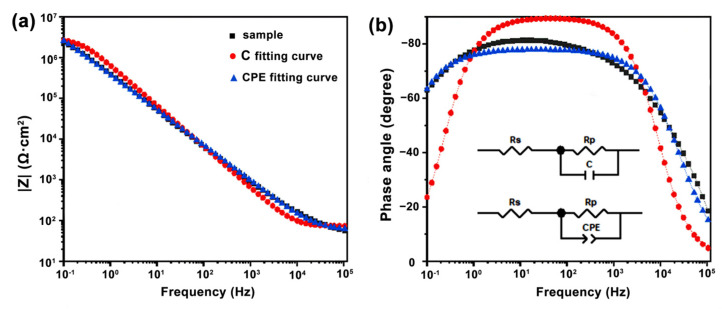
Electrochemical impedance spectra (**a**) and corresponding equivalent circuits (**b**) for the OFAC immersed in a 3.5 wt.% NaCl solution.

Analysis of the equivalent circuit diagrams (Figure 7) reveals that as immersion time increases, the capacitance of the sample rises while its resistance declines. This trend suggests a progressive infiltration of the electrolyte solution into the sample, which enhances charge transfer between the electrode and the electrolyte. Despite this general pattern, a significant discrepancy is observed between OFAC and Sample 14 at the same immersion time, particularly after 20 h. The capacitance and resistance values of sample 14 differ by an order of magnitude from those of OFAC, indicating that electrolyte penetration is less pronounced in OFAC. These differences imply that OFAC possesses a higher surface density, a more uniform surface structure, and fewer defects, all of which contribute to its superior water resistance. Specifically, the coating’s water repellency is primarily affected by surface roughness and surface energy. The introduction of nano-scale particles (additive B) creates a micro-nano structure on the surface, increasing roughness and thereby enhancing hydrophobicity. This structure reduces the contact area between water droplets and the coating surface, allowing the droplets to roll off more easily [31]. Additionally, the incorporation of water-repelling reagents (additive A) significantly lowers the surface energy of the coating, further improving its water-repellent properties. The reduction in surface energy decreases the adhesive force between water and the coating surface [32]. To sum up, in this section, we test the EIS of coatings. Through the EIS results, we verified the superior water-repellent performance of OFAC. Samples 12 and 10 initially have high impedance, but this decreases over time, indicating water infiltration and reduced water resistance. Sample 14 starts at an excellent resistance, but its impedance drops after 2 h, suggesting electrolyte penetration. The equivalent circuit diagrams reveal that capacitance increases and resistance decreases with longer immersion, indicating electrolyte infiltration. OFAC shows significantly lower permeability than sample 14, suggesting superior water resistance due to a higher surface density and fewer defects.

### 3.3. Condensation Performance

**Figure 8:**
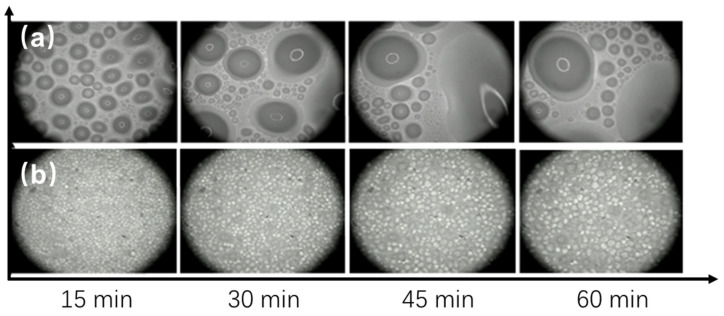
Time-lapse images of condensation behavior of the two coatings at a sub-cooled temperature of 9.4 °C observed under 50× magnification: (**a**) commercial fluorocarbon paint; (**b**) OFAC.

The condensation performance of the coatings was investigated by recording the condensation process in a custom-built environmental chamber. The effectiveness of the anti-condensation coating was further evaluated by comparing it with a commercial fluorocarbon coating. Figure 8 depicts the condensation behavior of the coatings at a sub-cooled temperature of 9.4 °C. Figure 8a shows time-lapse images of the commercial fluorocarbon paint on the Peltier cooling plate surface. Water droplets were observed almost immediately, indicating a high number of nucleation sites on the surface. As the test progressed, after 15 min, surface reflectivity increased significantly as small droplets formed randomly and gradually coalesced into larger ones. By 45 min, a continuous water film had developed, leading to stronger reflectivity. Shortly afterward, new droplets formed between the existing water film, merging to create an even larger film. In contrast, the coating of OFAC (Figure 8b), developed in this study, displayed a different condensation behavior. After 15 min, densely packed circular droplets formed on the surface. Image analysis revealed that most of these droplets had diameters ranging from 100 to 300 μm. As condensation continued, the droplets grew to approximately 600 μm, merging with neighboring droplets to form larger ones. The fusion of droplets left dry areas on the surface near the larger droplets, providing nucleation sites for new droplets, which initiated another growth cycle. This process occurred over a longer period compared to the commercial coating, with slower droplet growth and smaller overall droplet sizes. After merging, the smaller droplets retracted to a stable position, maintaining a large static contact angle.

**Figure 9:**
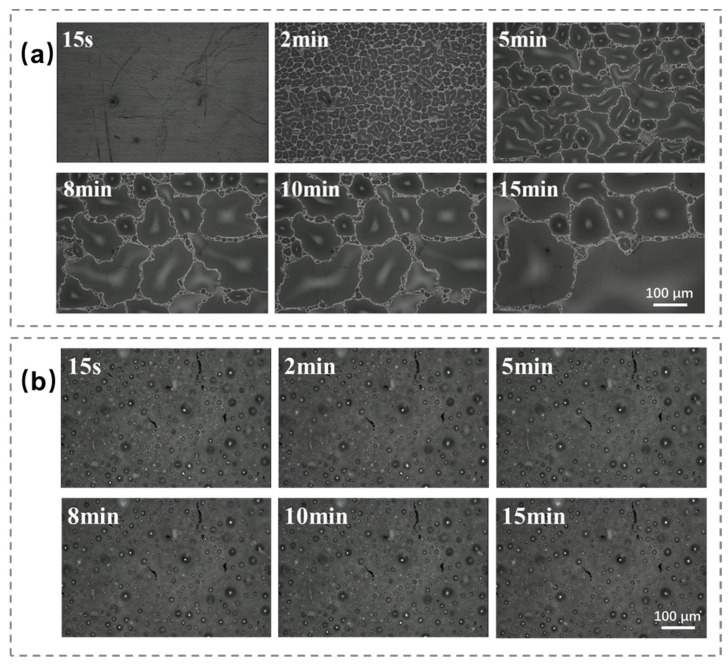
Condensation behavior of the two coatings observed under 200× magnification during the first 15 min: (**a**) commercial fluorocarbon paint; (**b**) OFAC.

A further investigation into the condensation process during the first 15 min was conducted using a metallographic microscope with 200× magnification, as shown in Figure 9. The development of the condensation process revealed that droplets on the surface of the commercial fluorocarbon coating were irregular during the nucleation stage, forming various shapes (Figure 9a). Over time, these irregular droplets gradually merged into a large water film, indicating that condensation on the commercial coating followed a film condensation pattern rather than droplet condensation. This behavior is attributed to the coating’s hydrophilicity. Higher surface energy increases hydrophilicity, promoting film condensation. The water film, once formed, adheres strongly to the surface, making it difficult to remove. As condensation progresses, this film grows and eventually compromises the insulation and waterproof properties of the coating. Generally, condensation is a dynamic cyclic process involving nucleation, growth, coalescence, and eventual detachment. Droplet separation marks the end of one cycle but is immediately followed by a new cycle, where droplets grow close to the nucleation site. As droplets grow beyond a certain size, they merge with adjacent droplets until they exceed the critical surface energy. After 15 min of cooling, the combined large water droplets have covered most of the surface area of the coated surface and gradually appear like a film.

In contrast, OFAC exhibited a delayed onset of droplet formation. On the commercial coating, condensation droplets appeared within 8 s, whereas in OFAC, sporadic droplets formed only after 15 s (Figure 9b). These droplets were small and scattered randomly across the coating surface, with a relatively slow growth rate. The droplet coalescence process typically occurred after 15 min. The wetting state is closely linked to the surface free energy [33]. On a hydrophobic surface with a high static contact angle, the droplet penetration is shallow, generally remaining in a Cassie-wetting state [34,35]. Additionally, droplets on the hydrophobic surface exhibit a pronounced “auto-shrink” behavior, which helps them maintain a stable radius. However, the heat transfer rate of individual droplets is minimal, which further delays droplet growth [36]. As a result, more time is required for the droplet to reach a critical size that disrupts the balance between internal forces and surface energy. Once this threshold is crossed, the droplets detach from the surface and roll away, exhibiting a self-cleaning or “self-drying” effect [37], which creates new nucleation sites for further condensation.

## 4. Conclusions

A hydrophobic coating was successfully developed using an orthogonal design approach combined with a simple spray method. The goal of this approach was to create an anti-condensation coating with a micro-nano structure, providing enhanced water resistance. Optical microscopy observations of condensation nucleation and growth on the coating’s surface revealed a delayed onset of droplet formation and smaller droplet sizes compared to non-coated surfaces. These findings demonstrate that the micro-nano structured coating with low surface energy effectively suppresses and delays the condensation process, reducing the risk of damage from moisture accumulation following the condensation mechanism. Consequently, the anti-condensation surface developed in this study offers a novel strategy for designing low-surface energy coatings while also broadening their potential for industrial applications.

## Figures and Tables

**Table 1:** Influencing factors and levels of orthogonal experiment.

Factor	A	B	C	D	E	F
	Water-Repelling Reagent (g)	Nanofiller (g)	Antistatic Reagent (g)	Anti-Mold and Anti-Algae Reagent (g)	Levelling Reagent (g)	Wetting and Dispersing Reagent (g)
1	0.3	2	1	0.3	0.3	0.3
2	0.6	4	2	0.6	0.6	0.6
3	0.9	6	3	0.9	0.9	0.9

**Table 2:** Orthogonal experiment scheme.

Sample No.	A	B	C	D	E	F
	Water-Repelling Reagent (g)	Nanofiller (g)	Antistatic Reagent (g)	Anti-Mold and Anti-Algae Reagent (g)	Levelling Reagent (g)	Wetting and Dispersing Reagent (g)
1	0.3	4	3	0.9	0.9	0.6
2	0.6	6	1	0.6	0.9	0.3
3	0.6	4	3	0.6	0.6	0.3
4	0.9	4	2	0.6	0.3	0.9
5	0.3	6	1	0.9	0.6	0.9
6	0.3	6	3	0.6	0.3	0.9
7	0.9	6	2	0.9	0.9	0.3
8	0.9	2	1	0.6	0.6	0.6
9	0.9	2	3	0.9	0.3	0.3
10	0.6	4	1	0.9	0.3	0.6
11	0.9	4	1	0.3	0.9	0.9
12	0.3	2	1	0.3	0.3	0.3
13	0.3	2	2	0.6	0.9	0.6
14	0.9	6	3	0.3	0.6	0.6
15	0.6	2	3	0.3	0.9	0.9
16	0.6	6	2	0.3	0.3	0.6
17	0.3	4	2	0.3	0.6	0.3
18	0.6	2	2	0.9	0.6	0.9

**Table 3:** Test liquids (mN/m).

Liquid	γ	$\gamma^{LW}$	$\gamma^{+}$	$\gamma^{-}$
Water	72.8	21.8	25.5	25.5
Formamide	58.0	39.0	2.28	39.6
Diiodomethane	50.8	50.8	≈0	≈0

**Table 4:** Contact angle and surface energy.

Sample	Contact Angle (°)			Surface Energy (γ/mJ·m^−2^)
	Water	Formamide	Diiodomethane	
1	110	94	67	26.40
2	121	87	73	22.88
3	117	90	73	21.36
4	122	93	77	19.51
5	105	84	69	23.59
6	101	82	68	24.24
7	124	97	78	18.59
8	118	90	70	22.88
9	118	90	74	20.98
10	112	88	70	22.87
11	100	85	70	23.33
12	95	80	65	26.10
13	102	93	71	26.01
14	125	97	79	19.83
15	114	91	72	21.78
16	116	91	72	21.78
17	105	94	70	26.12
18	108	95	73	23.23

**Table 5:** Range analysis results of the surface energy in the orthogonal experiment.

Horizontal Mean	A	B	C	D	E	F
	Water-Repelling Reagent (g)	Nanofiller (g)	Antistatic Reagent (g)	Anti-Mold and Anti-Algae Reagent	Levelling Reagent	Wetting and Dispersing Reagent
Mean 1	25.41036	23.49698	23.60835	23.1578	22.57994	22.67107
Mean 2	22.31752	23.26494	22.54105	22.81377	22.83516	23.29596
Mean 3	20.85295	21.81891	22.43144	22.60926	23.16573	22.61379
Range	4.557	1.678	1.177	0.549	0.586	0.682

**Table 6:** OFAC surface wettability test.

/	Contact Angle (°)			$\gamma^{LW}$	$\gamma^{+}$	$\gamma^{-}$	Surface Energy (γ/mJ·m^−2^)
	Water	Formamide	Diiodomethane				
OFAC	136	102	85	15.01	0.06	3.22	15.86

## Data Availability

The original contributions presented in the study are included in the article, further inquiries can be directed to the corresponding author.
